# Structural and Thermal Investigations of Co(II) and Ni(II) Coordination Polymers Based on biphenyl-4,4′-dioxydiacetate Linker

**DOI:** 10.3390/ma14133545

**Published:** 2021-06-25

**Authors:** Halina Głuchowska, Renata Łyszczek, Liliana Mazur, Alexander M. Kirillov

**Affiliations:** 1 Department of General and Coordination Chemistry and Crystallography, Faculty of Chemistry, Institute of Chemical Sciences, Maria Curie-Skłodowska University in Lublin, M.C. Skłodowskiej Sq. 2, 20-031 Lublin, Poland; liliana.mazur@poczta.umcs.lublin.pl; 2 Centro de Química Estrutural, Departamento de Engenharia Química, Instituto Superior Técnico, Universidade de Lisboa, Av. Rovisco Pais, 1049-001 Lisbon, Portugal; kirillov@tecnico.ulisboa.pt; 3 Research Institute of Chemistry, Peoples’ Friendship University of Russia (RUDN University), 6 Miklukho-Maklaya st., 117198 Moscow, Russia

**Keywords:** coordination polymers, crystal structure, infrared spectra, thermal analysis, TG-FTIR

## Abstract

Two coordination polymers, [Co(µ_4_-L)(H_2_O)_2_]*_n_* (**1**) and [Ni(µ-L)(H_2_O)_4_]*_n_* (**2**), were solvothermally assembled from the corresponding metal(II) chlorides and biphenyl-4,4-dioxydiacetic acid (H_2_L) as a flexible dicarboxylate linker. The cobalt(II) compound **1** featured a layer-pillared 3D metal-organic network with a cds topology, while the nickel(II) derivative **2** represented a linear chain 1D coordination polymer with a 2C1 topology. The µ_4−_ and µ-L^2−^ linkers exhibited different denticity and coordination modes in the synthesized compounds, thus contributing to their structural diversity. The dimensionality of **1** and **2** had an influence on their thermal stability and decomposition processes, which were investigated in detail by TG-DSC and TG-FTIR methods. Thermal decomposition products of coordination polymers were also analyzed by PXRD, confirming the formation of Co_3_O_4_/CoO and NiO as final materials. The obtained compounds broaden a family of coordination polymers assembled from flexible dicarboxylate linkers.

## 1. Introduction

Coordination polymers represent a very attractive generation of functional materials due to their prominent physico-chemical properties, which arise from the combination of inorganic and organic building blocks that are joined via strong covalent bonds into the infinite one-, two- or three-dimensional structures [1,2,3,4,5]. These materials exhibit a great potential for applications in many areas, including storage and separation of gases and small molecules, catalysis, chemical sensors, medicine, and magnetic and photonic materials [6,7,8,9,10,11,12,13,14,15,16,17].

Multidentate ligands, derived from aromatic polycarboxylic acids, are commonly employed as organic building blocks due to the rich coordination modes of carboxylate groups, which strongly impact the structural diversity and topologies of polymeric architecture and their functionality. The energy of the metal-oxygen bonds in carboxylate complexes (ca. 200–400 kJ/mol) is of greater value than the energy of metal-nitrogen bonds in coordination compounds containing neutral ligands (ca. 60–180 kJ/mol) [18,19,20,21,22]. The linking abilities of carboxylate ligands are also attributed to the privileged arrangement of their COO groups. 1,4-Benzenedicarboxylic acid, and its expanded analogues, are extensively used in the construction of different types of coordination polymers with large pore sizes and an array of functional properties [1,2,3,4,23,24,25,26,27]. Apart from the polycarboxylic acid linkers that comprise a rigid biphenyl, naphthalene, pyrene, or pyrazine moiety, diverse flexible multicarboxylate linkers have gained particular attention for generating novel metal-organic architectures [28,29,30,31,32].

**Scheme 1:**
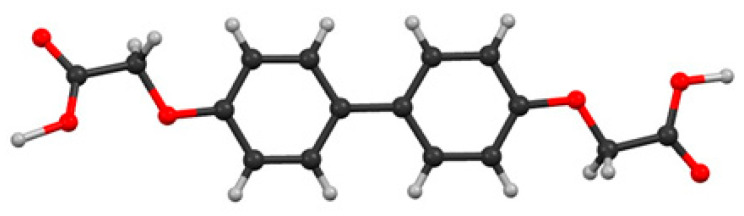
Structure of biphenyl-4,4’-dioxydiacetic acid (H_2_L).

The present study is a continuation of our research on coordination polymers assembled from aromatic polycarboxylic acids [33,34,35,36,37,38]. Recently, our attention has focused on probing biphenyl-4,4’-dioxydiacetic acid (H_2_L) (Scheme 1) as a flexible linker toward lanthanides and alkali metals [39,40]. This linker offers up to six sites (four carboxylate and two ether) for potential coordination, and possesses good thermal stability and conformational versatility [39,40,41]. It was also postulated that the ionic radius of a metal center might be a main factor that influences the denticity of ligand and dimensionality of the self-assembled coordination polymers. For example, in the lithium derivative, metal centers are coordinated by only carboxylate oxygen atoms, while in the Na- and K-containing compounds, the side-chain oxygen atoms of H_2_L are also involved in the coordination. Some coordination compounds of biphenyl-4,4’-dioxydiacetic acid, with selected transition metals, were reported by Ji et al. [41]. It was shown that the dicarboxylate ligand is bound to Co(II) and Ni(II) ions through carboxylate groups, forming the isostructural one-dimensional coordination polymers, while the Cd(II) ions are additionally bound by O-ether functionalities generating the two-dimensional layers [41].

With an aim to demonstrate a coordination diversity of H_2_L toward metal ions with very similar ionic radii, herein we will report on the synthesis and detailed structural investigation of two coordination polymers, namely [Co(µ_4_-L)(H_2_O)_2_]*_n_* (**1**) and [Ni(µ-L)(H_2_O)_4_]*_n_* (**2**). Apart from possessing similar ionic radii, the selection of Co(II) and Ni(II) in the present work was governed by their abundance, low cost, versatile coordination chemistry with carboxylate ligands, and the variety of functional applications of such products and derived materials [42]. Additionally, simultaneous reports on cobalt(II) and nickel(II) coordination compounds bearing related types of flexible dicarboxylate ligands are scant. A particular focus of this research was also on the thermal behavior of synthesized compounds in air and nitrogen atmospheres, accounting for the fact that these coordination polymers can be precursors of transition metal oxide materials [43,44]. We also examined the thermal stability and decomposition processes of **1** and **2** using TG-DSC and TG-FTIR methods.

## 2. Materials and Methods

Biphenyl-4,4’-dioxydiacetic acid (H_2_L) was prepared according to the literature procedure [40]. The metal sources CoCl_2_·6H_2_O and NiCl_2_·6H_2_O, as well as N,N-dimethylformamide (DMF), were purchased from Alfa-Aesar and used without further purification.

### 2.1. Synthesis of Coordination Polymers and Preparation of Metal Oxides

[Co(µ_4_-L)(H_2_O)_2_]_n_ (**1**). CoCl_2_·6H_2_O (237.9 mg, 1 mmol) and H_2_L (302 mg, 1 mmol) were dissolved in a mixture of solvents (25 mL of DMF and 25 mL of deionized water) and then transferred into the Teflon-lined stainless steel autoclave (150 mL volume). This was sealed and heated under solvothermal conditions at 120 °C over 72 h. The autoclave was then gradually cooled down to room temperature (5 °C/h cooling rate). The pink crystals were filtered off, washed with a water and DMF mixture, and dried at room temperature to give the compound **1** (yield: 76% based on cobalt(II) chloride). Anal. Calc. for **1**: C, 48.58; H, 4.05. Found: C, 48.21; H, 4.01%.

[Ni(µ-L)(H_2_O)_4_]_n_ (**2**). This compound was obtained by following a procedure similar to **1**, except using NiCl_2_·6H_2_O (238 mg, 1 mmol) instead of CoCl_2_·6H_2_O. The green crystals were filtered off, washed with a water and DMF mixture, and dried at room temperature to give the compound **2** (yield: 68% based on nickel(II) chloride). Anal. Calc. for **2**: C, 44.54; H, 4.64. Found: C, 44.12; H, 4.23%.

The samples of coordination polymers (100 mg) were heated for 2 h in ceramic crucibles in an electric furnace up to 1000 °C in a static air atmosphere. Metal oxides were obtained in the form of black powders and analyzed by PXRD.

### 2.2. Materials Characterization

The contents of C and H in the prepared compounds were determined by elemental analysis with a EuroEA3000 elemental analyzer (EuroVector S.p.A., Milan, Italy).

The ATR-FTIR (attenuated total reflection–Fourier transform infrared spectroscopy) spectra were recorded on a Nicolet 6700 spectrophotometer equipped with the Smart iTR attachment (diamond crystal) over 4000–600 cm^−1^ (Thermo Scientific, Waltham, MA, USA).

Thermal analyses were carried out by the TG-DSC methods using a Setsys 16/18 analyzer (Setaram, Caluire, France). The samples (~8 mg) were heated in alumina crucibles in the flowing air atmosphere (*v* = 0.75 dm^3^ h^−1^) at a heating rate of 10 °C min^−1^ in the 30–1000 °C range.

The FTIR (Fourier transform infrared spectroscopy) spectra of volatile products, formed during the decomposition of coordination polymers, were measured using a Q5000 TA apparatus (TA Instruments, New Castle, DE, USA) coupled with the Nicolet 6700 FTIR spectrophotometer.The samples (~20 mg) were heated in open platinum crucibles up to 700 °C at a heating rate of 20 °C min^−1^ in a flowing nitrogen atmosphere (25 cm^3^ min^−1^).

The PXRD (powder X-ray diffraction) patterns of the synthesized coordination compounds and metal oxides were recorded at room temperature using a PANalytical Empyrean (Panalytical, Almelo, Netherlands) diffractometer (Bragg-Brentano method, *Cu*-Kα radiation). The data were collected in the 5–90° range with a step size of 0.05°.

### 2.3. Single Crystal X-ray Diffraction

The X-ray diffraction data were collected at 100(2) K using an Oxford Diffraction Xcalibur CCD diffractometer (Oxford Diffraction Ltd., Abingdon, UK) equipped with a molybdenum sealed X-ray tube (*λ* = 0.7071 Å), a graphite monochromator, and an Oxford Cryosystems nitrogen gas flow device (Cobra Plus). The CrysAlis [45] suite of programs was used for data collection, cell refinement, and data reduction. The data were corrected for Lorentz and polarization effects. A multi-scan absorption correction was applied. The structures were solved using direct methods (SHELXS-97 program) and refined by the full-matrix least squares on *F*^2^ with the SHELXL-97 program [46]. All non-H atoms were refined with anisotropic displacement parameters. The H atoms attached to carbon centers were positioned geometrically and refined using a riding model with *U*_iso_(H) = 1.2*U*_eq_(C). The water H atoms were found in the difference-Fourier map and refined with isotropic displacement parameters. Crystal data and structure refinement details for **1** and **2** are given in Table 1. The CIF files for **1** and **2** were deposited at the Cambridge Crystallographic Data Centre (CCDC Nos 2073788–2073789).

### 2.4. Topological Analysis

To get further insight into the crystal structures of **1** and **2**, we performed their topological analysis by applying the underlying network concept [47,48,49,50]. The underlying nets were generated by omitting all the terminal H_2_O ligands and reducing the µ-L^2−^ or µ_4_-L^2−^ linkers to the corresponding centroids, preserving their connectivity with metal nodes.

## 3. Results

The reactions of cobalt(II) or nickel(II) chloride with biphenyl-4,4*′*-dioxydiacetic acid (H_2_L) under solvothermal conditions afforded three-dimensional [Co(µ_4_-L)(H_2_O)_2_]_n_ (**1**) and one-dimensional [Ni(µ-L)(H_2_O)_4_]_n_ (**2**) coordination polymers. The powder X-ray diffraction patterns of the obtained products fit well with the simulated ones (Figures S1 and S2), thus confirming a purity of the synthesized samples. The same solvothermal conditions furnish a unique combination of solvents (DMF/H_2_O, 1:1), temperature (120 °C), time of heating (72 h), and pressure (autogenous) for the generation and crystallization of such compounds. A related 1D Co(II) coordination polymer [41] was synthesized under following synthetic conditions: DMF/H_2_O 1:4, 85 °C, and 48 h. A change in the synthesis parameters led to the formation of the three-dimensional coordination polymer **1**. On the other hand, a nickel(II) complex [41] was prepared using a solvent mixture (DMF/H_2_O, 1:1), as in the synthesis of **2**, while temperature and time were different (80 °C, 24 h). Both sets of parameters affected the formation of products, suggesting that the solvent composition and nature of metal ions were the dominant factors that influenced the dimensionality and structural type of coordination polymers.

Both solvents play crucial, but different, functions during the formation of **1** and **2**. In fact, DMF is an excellent polar solvent for deprotonation of H_2_L [51]. On the other hand, the presence of water is not restricted to a role of solvent since it also acts as a ligand. Despite a similarity of the reaction conditions, the compounds **1** and **2** featured distinct 3D (three-dimensional) and 1D (one-dimensional) coordination polymer networks, respectively, thus confirming a structure-defining role of metal(II) centers. In both compounds, the coordination environment of metal building units can originate from soluble mononuclear hexaaqua metal(II) complexes, which are initially formed during the dissolution of metal precursors [52,53]. The formation of the coordination polymers with the biphenyl-4,4’-dioxydiacetate linkers can be regarded as a partial substitution of aqua ligands by organic dicarboxylate ones.

### 3.1. Crystal Structure Description

**Figure 1:**
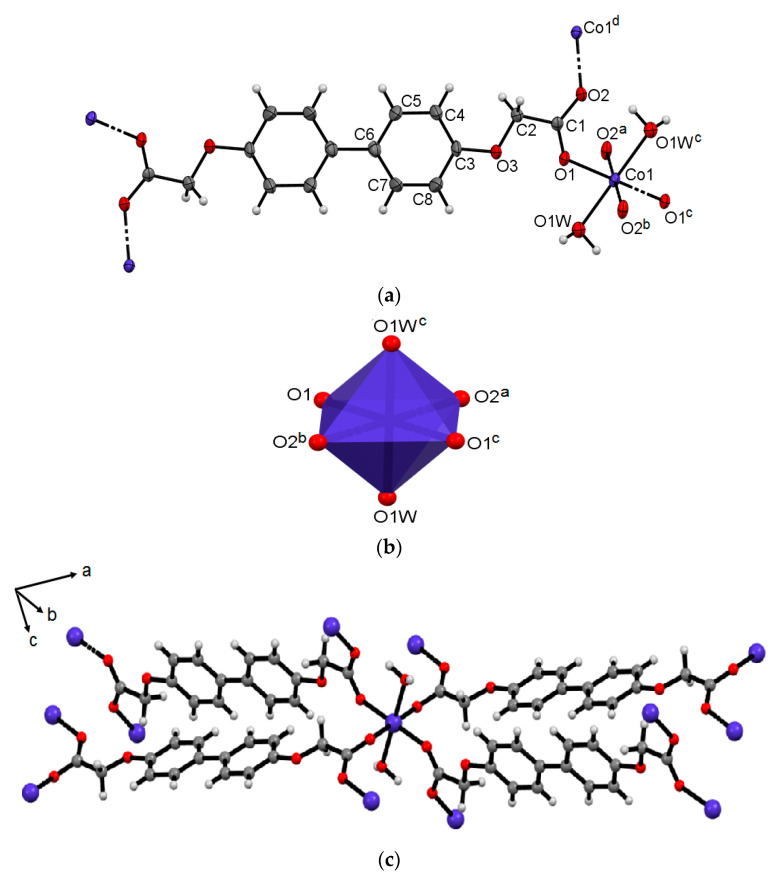

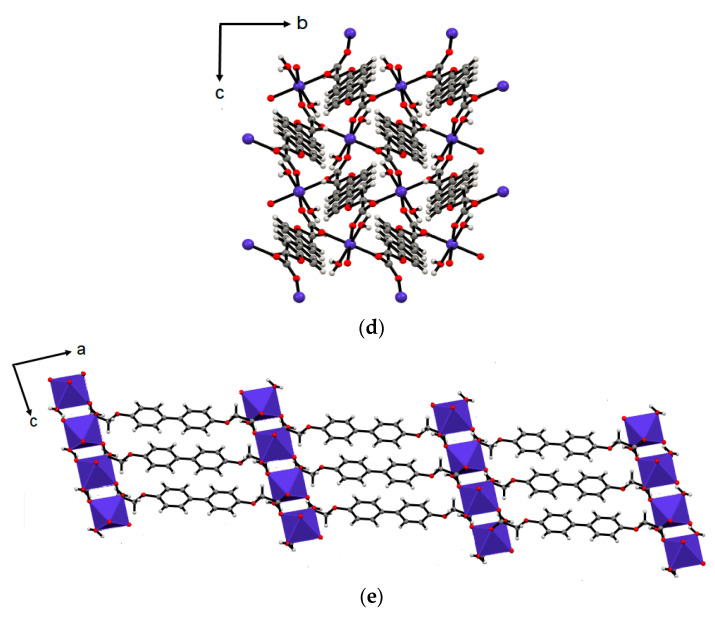
Structural fragments of **1**. (**a**) Coordination environment of Co center; 50% probability level ellipsoids are drawn; (**b**) coordination polyhedron of Co atom; (**c**) ligand arrangement around metal center; (**d**) 2D layer motif in view along the *a* axis; and (**e**) 3D metal-organic network with polyhedral representation of Co atoms; view along the *b* axis. Symmetry codes: (a) x, −y + 0.5, z + 0.5; (b) –x, y−0.5, −z + 0.5; (c) –x, −z + 1, −z + 1; and (d) −x + 2, −y + 1, −z + 1.

[Co(µ_4_-L)(H_2_O)_2_]_n_ (**1**). This 3D coordination polymer (Figure 1) crystallizes in the monoclinic *P*2_1_/*c* space group. The asymmetric unit comprises of half cobalt(II) ion, half µ_4_-L^2−^ linker, and one terminal H_2_O ligand. The Co1 coordination environment contained four carboxylate oxygen atoms from four µ_4_-L^2−^ blocks and two oxygen atoms from *trans* aqua ligands (Figure 1a). Such an arrangement of cobalt (II) center is relatively common in carboxylate-based coordination compounds obtained in an aqueous medium [42,54].

The hexa-coordinated cobalt(II) center adopted an almost ideal octahedral environment with carboxylate oxygen atoms in basal sites and water molecules in axial positions (Figure 1b). The Co–O_carb_ bond distances were 2.078(2) and 2.068(2) Å (Table 2), while the Co–O_W_ bond was slightly longer (2.133(2) Å) due to the Jahn-Teller distortion [55]. The O–Co–O angles, which should be 90° in a regular octahedral arrangement, ranged from 81.90(8) to 98.10(8)°, whereas the remaining angles were exactly 180° (Table 2).

The biphenyl-4,4’-dioxydiacetate ligand acted as a tetradentate μ_4_-spacer bridging four cobalt (II) centers through both carboxylate groups (Figure 1a,c). The mutually *trans* µ_4_-L^2−^ linkers were aligned parallel to each other (Figure 1c), while those that are mutually *cis* were rotated by 77.48° to each other. The angles of carboxylate groups in µ_4_-L^2−^ had a typical value of 124.6(3)°, which is characteristic for the bidentate-bridging COO^−^ mode [42]. Carboxylate groups bind cobalt atoms in a non-planar *syn-anti* mode and both carboxylate O atoms were slightly displaced from the plane of the –O–CH_2_–C– moiety (O2–C1–C2–O3 and O1–C1–C2–O3 torsion angles were 167.7(2) and −13.0(4)°, respectively). The oxyacetate arms –O–CH_2_–COO of the ligand were deviated from the plane of an almost planar biphenyl ring (torsion angles: C2–O3–C3–C8 175.4(3)°; and C2–O3–C3–C4 −3.2(5)°). The adjacent Co1 centers were arranged into 2D (two-dimensional) layer cobalt-carboxylate motifs (Figure 1d) that were further interlinked, via the remaining carboxylate functionalities of µ_4_-L^2−^, into a 3D layer-pillared framework (Figure 1e). The structure was also stabilized by a network of hydrogen bonds (Table S1). The aqua ligands took part in four hydrogen bonds, with the O···O distances ranging from 2.681(3) to 3.052(3) Å, acting as both proton donors and acceptors. It is worth mentioning that both water ligands participated in bifurcated hydrogen bonds. Carboxylate oxygen atoms and oxygen from the side chain played the role of proton acceptors in H-bonding interactions (Figure S3).

**Figure 2:**
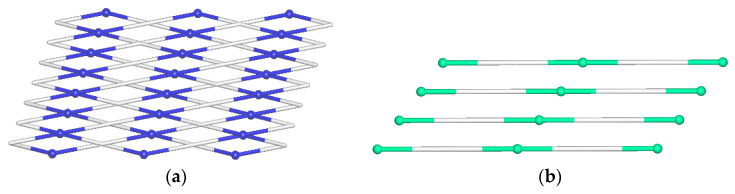
Topological representations of simplified 3D (**a**) and 1D (**b**) metal-organic networks in **1** and **2**; views along the b axis. The (**a**) mononodal 4-connected net in **1** with a cds topology; 4-connected Co nodes (blue balls), and centroids of 4-connected µ_4_-L^2−^ linkers (gray); and (**b**) four linear chains showing a 2-linked net in **2** with a 2C1 topology; Ni nodes (green balls), and centroids of µ-L^2−^ linkers (gray).

The 3D structure of **1** was also analyzed from a topological viewpoint (Figure 2). An intricate 3D metal-organic architecture was driven by the [Co(H_2_O)_2_]^2+^ blocks and the µ_4_-L^2−^ linkers. Both of these structural blocks are considered as 4-linked nodes that are topologically similar. Hence, an underlying framework in **1** can be classified as a mononodal 4-linked net with a cds (CdSO_4_) topology and the point symbol of (6^5^.8) (Figure 2a) [56,57].

**Figure 3:**
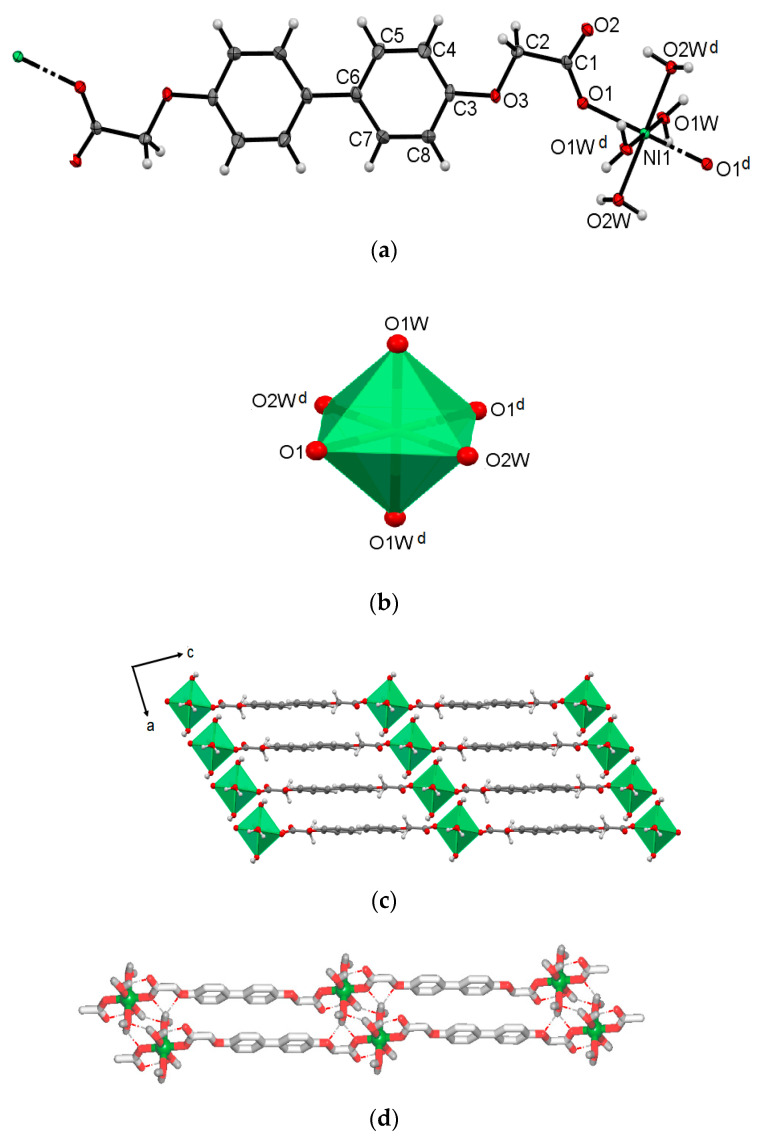
Structural fragments of **2**. (**a**) Coordination environment of Ni center; 50% probability level ellipsoids are drawn; (**b**) coordination polyhedron of Ni atom; and (**c**) crystal packing with polyhedral representation of Ni atoms in view along the *b* axis. Symmetry code: (d) –x + 2, −y + 1, z + 1. (**d**) H-bond network (dotted lines) in compound **2**.

[Ni(µ-L)(H_2_O)_4_]_n_ (**2**). This 1D coordination polymer (Figure 3) crystallized in the triclinic *P*-1 space group with half of the metal ion, half of the dicarboxylate ligand, and two terminal water ligands in the asymmetric unit. This compound is isostructural with the previously reported one-dimensional biphenyl-4,4’-diacetate prepared under different conditions [41]. Two carboxylate oxygen atoms from different µ-L^2−^ linkers and four water molecules formed an almost ideal octahedron around the Ni1 center (Figure 3a,b). The Ni–O_carb_ bond lengths were 2.051(2) Å, while the Ni–O_W_ distances were 2.032(2) and 2.073(2) Å. The angles that should be 90° in the regular octahedron ranged from 87.35(7) to 92.65(7)°; the remaining angles were 180° (Figure 3b). The bond lengths and angles in the coordination octahedron were within those observed in a related nickel compound [41]. The µ-L^2−^ ligand acted as a bidentate linker to form a 1D linear chain structure (Figure 3c), as observed in related compounds [40,41]. Both carboxylate groups were monodentate with the O2–C1–O1 angle equal to 126.5(2)°. The biphenyl ring and side arms of µ-L^2−^ were almost coplanar. As depicted in Figure 3d and Figure S4, there was an extensive network of intramolecular and intermolecular hydrogen bonds (Table S1), with the latter responsible for a structural extension into a 3D H-bonded network. Water molecules participated in the bifurcated hydrogen bonds: three times as proton donor and one as proton acceptor. Topological analysis of **2** classified the 1D metal-organic chains within a 2C1 type (Figure 2b) [58,59], while the 3D H-bonded net featured a 6,8T13 topology.

### 3.2. Analysis of Infrared Spectra

**Figure 4:**
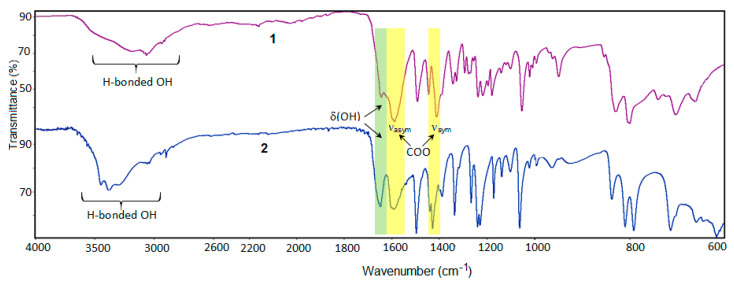
Infrared spectra of **1** and **2**.

The infrared spectra of **1** and **2** featured absorption bands due to the OH groups from the water ligands in the stretching vibration region of 3500–3000 cm^−1^, with maxima at 3376 and 3381 cm^−1^, respectively (Figure 4). An involvement of H_2_O ligands in hydrogen bonds results in the band broadening [60,61]. The presence of coordinated water molecules in both **1** and **2** gave rise to the appearance of strong bending δ(OH) vibrations at 1648 and 1651 cm^−1^, respectively [62]. Infrared spectra were also dominated by asymmetric and symmetric stretching vibrations of carboxylate groups, which appeared at 1601/1428 and 1592/1429 cm^−1^ for **1** and **2**, respectively. Slightly different positions of bands arising from asymmetric stretching vibrations of COO^−^ groups confirmed the different coordination modes in **1** and **2** [63].

### 3.3. Thermal Analysis in Air

**Figure 5:**
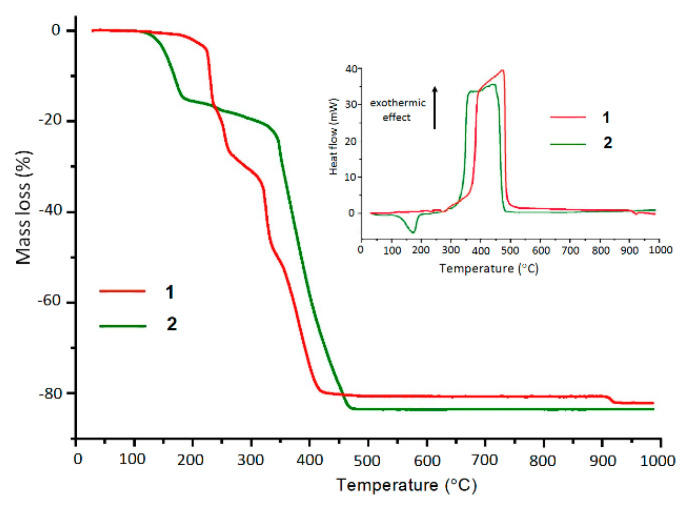
TG curves of **1** and **2** recorded in air atmosphere. The inset shows DSC curves.

The investigated compounds exhibited different thermal behavior according to the thermogravimetric (TG) and differential scanning calorimetry (DSC) analyses in an air atmosphere. The TG curve of **1** showed that the compound was stable up to 230 °C (Figure 5), followed by a multistep decomposition process connected with a release of water molecules and the burning of organic ligands, as attested by a broad exothermic effect. The degradation of metal-organic framework, with a mass loss of 78%, was nearly complete at 480 °C. At first, Co_3_O_4_ was formed as a solid residue of **1** under heating in air. This product was partially transferred into CoO in the temperature range of 900–940 °C. This process was accompanied by a minor endothermic effect on the DSC curve [64].

The 1D coordination polymer **2** showed lower thermal stability in comparison with **1** (Figure 5). The first mass loss (17.10%), observed on the TG curve at 150–200 °C, was ascribed to the release of four aqua ligands (calcd. 16.70%). The DSC profile indicated that the dehydration process took place in one step with an endothermic effect at 170 °C (peak top). A dehydrated sample was unstable on further heating, resulting in two-stage decomposition in the temperature ranges of 210–360 and 361–470 °C with mass losses of 5.37 and 59.70%, respectively. This significant mass loss corresponds to the burning of the organic ligand and the formation of NiO as a final solid residue [65].

### 3.4. PXRD Analysis of Metal Oxides

**Figure 6:**
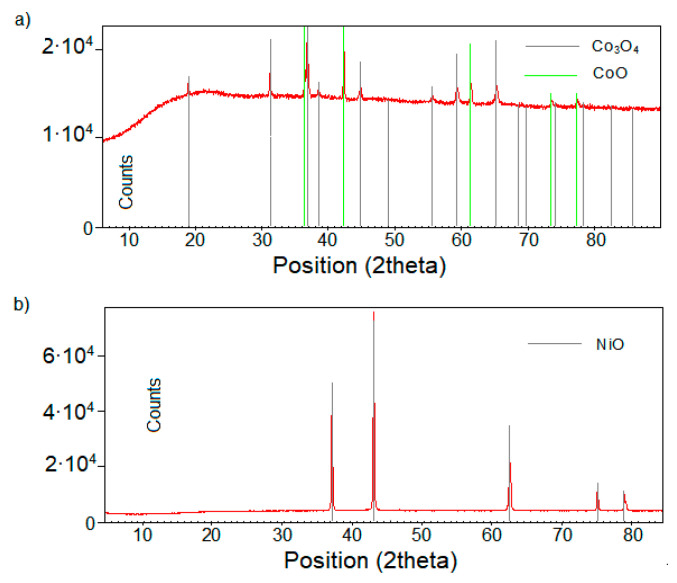
PXRD patterns of cobalt oxides (**a**) and (**b**) nickel oxide.

The PXRD patterns for the metal oxides (Figure 6), prepared on the basis of the compounds **1** and **2**, were compared with the reference data from the PDF ICDD database [66]. Heating of **1** at 1000 °C for 2 h gives a mixture of Co_3_O_4_ (65(1)%) and CoO (35(1)%), according to the reference data 04-005-7243 and 01-076-3830, respectively. Co_3_O_4_ is characterized by the following crystallographic data: cubic crystal system, space group F3d-3m, and unit cell parameters: *a* = *b* = *c* = 8.0920 Å, *Z* = 8, and *V* = 529.87 Å^3^. The crystallographic data of CoO are as follows: cubic crystal system, Fm-3m space group, and unit cell parameters: *a* = *b* = *c* = 4.7230 Å, *Z* = 4, and *V* = 78.02 Å^3^.

Heating of **2** led to the formation of NiO, which perfectly fits with the reference data 04-006-1894. This nickel(II) oxide crystallized in the cubic crystal system with the Fm-3m space group and unit cell parameters: *a* = *b* = *c* = 4.1800 Å, *Z* = 4, and *V* = 73.03 Å^3^.

### 3.5. TG-FTIR Analysis

**Figure 7:**
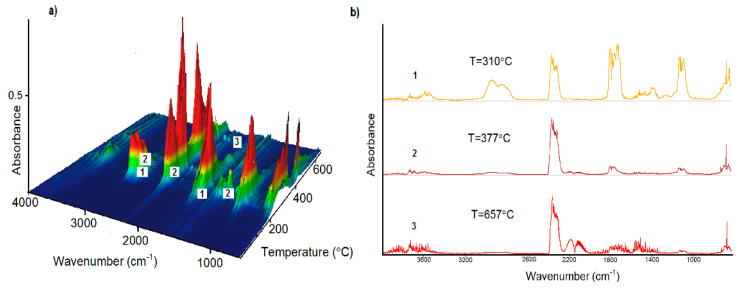
(**a**) 3D representation of FTIR spectra of evolved gases during decomposition of **1**; and (**b**) FTIR spectra of gaseous products recorded at different temperatures.

**Figure 8:**
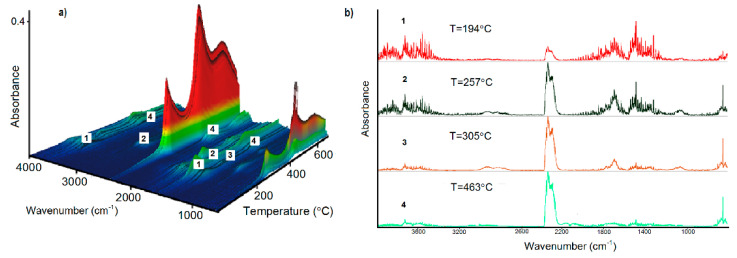
(**a**) 3D representation of FTIR spectra of evolved gases during the decomposition of **2**; and (**b**) FTIR spectra of gaseous products recorded at different temperatures.

To assess the thermal stability of **1** and **2** in an inert atmosphere, as well as to identify the gaseous products of their thermal decomposition, the coupling of thermogravimetric analysis (TG) and Fourier transform infrared spectroscopy (FTIR) was applied (Figure S5, Figure 7 and Figure 8).

The compound **1** is thermally stable in a nitrogen atmosphere up to 189 °C. During further heating, three stages of decomposition were observed in the temperature ranges of 190–308, 309–378, and 379–600 °C, which were accompanied by mass losses of 32.9, 13.4, and 21.2%, respectively. As can be deduced from the infrared spectra of volatile products evolved during the heating of **1** under nitrogen, the dehydration took place along with the decomposition process. The molecules of water, formic acid, and carbon dioxide were released at first (Figure 7). The FTIR spectra showed weak broad bands in the wavenumber ranges of 4000–3400 and 1900–1300 cm^−1^ as a result of stretching and deformation vibrations of OH groups from evolved water molecules [33,34,35,36,37,38]. In the range of 3150–2600 cm^−1^, there was a broad band with the maxima at 2931 and 2854 cm^−1^ due to the stretching CH vibrations from evolved formic acid [67]. Several bands at 1792, 1749, and 1723 cm^−1^ were attributed to the stretching vibrations of the carbonyl group. The relatively strong bands, with maxima at 1121 and 1081 cm^−1^, were assigned to the COH groups from the evolved HCOOH molecules. The highest intensities of such gas evolution was observed up to about 400 °C. Carbon dioxide molecules gave characteristic bands in the 2400–2250 cm^−1^ range (maxima at 2358, 2344, 2321, and 2310 cm^−1^) and one band at 668 cm^−1^ due to the stretching and deformation vibrations. Intense carbon dioxide evolution was observed up to about 600 °C. Carbon monoxide molecules were also identified among the volatile products of the compound **1** decomposition (Figure 7), as attested by a very diagnostic double band in the 2250–2000 cm^−1^ range [33,34,35,36,37,38]. The most intense release of CO took place above 400 °C. It is worth mentioning that methane was also observed above 250 °C, as attested by a group of bands with a maximum at 3014 cm^−1^.

Compound **2** was thermally stable in a nitrogen atmosphere up to 102 °C. Then, an overlapping four-stage decomposition occurred in the temperature ranges of 103–203, 204–285, 286–486, and 487–700 °C, accompanied by mass losses of 12.2, 9, 30.3, and 5.9%, respectively (Figure S4). The compounds that were isostructural with **2** [41] exhibited different thermal behavior in a nitrogen atmosphere. Their dehydration process took place in the temperature range of 86–151 °C, with the formation of dehydrated complexes stable up to ~400 °C. These distinct thermal behaviors can be explained by supramolecular differences (H-bonding networks and other weak interactions), which have an effect on the solid state reactions during heating.

The water was the main volatile product observed in the temperature range of 105–245 °C, as can be proposed based on the FTIR spectra (Figure 8). Further heating led to the degradation of the compound and the evolution of such volatile products as carbon dioxide and formic acid. Additionally, some traces of carbon monoxide were also recognized. Above 420 °C, the increase of intensity of CO_2_ bands, as well as the evolution of CO, were observed. In comparison to the degradation of **1**, methane molecules were identified among gaseous products above 430 °C. The total mass loss associated with heating of **2** in nitrogen was 57.4%.

## 4. Conclusions

In this work, we further explored biphenyl-4,4’-dioxydiacetic acid (H_2_L) as a flexible dicarboxylate linker for the solvothermal assembly of cobalt(II) and nickel(II) coordination polymers. Both products [Co(µ_4_-L)(H_2_O)_2_]_n_ (**1**) and [Ni(µ-L)(H_2_O)_4_]_n_ (**2**) were structurally characterized. Despite a similarity in synthetic procedures, the compounds featured distinct 3D (**1**) and 1D (**2**) structures and topologies. The dimensionality of the obtained compounds had an influence on their thermal stability and decomposition processes, which were investigated in detail by TG-DSC and TG-FTIR methods. Thermal decomposition products of **1** and **2** were also analyzed by PXRD, confirming the formation of Co_3_O_4_/CoO and NiO. The coordination polymers were stable in air up to 230 °C (**1**) and 150 °C (**2**), and their decomposition proceeded with an evolution of water, formic acid, and carbon oxides as the main species. We believe this study contributes to the widening of a family of metal-organic architectures assembled from flexible carboxylate linkers and to the gathering of information on their thermal behavior with relevance to the synthesis of metal oxide materials. It should be noted that coordination polymers represent a very promising class of precursors for the generation of metal oxide nanoparticles and related composite materials with interesting morphological and electrochemical properties [68,69,70,71]. Further research in this direction is underway in our laboratories.

## Figures and Tables

**Table 1:** Crystallographic data and refinement details for compounds **1** and **2**.

Compound	1	2
Formula	CoC_16_H_16_O_8_	NiC_16_H_20_O_10_
Molecular weight	395.22	431.03
*T* (K)	100(2)	100(2)
Crystal system	monoclinic	triclinic
Space group	*P*2_1_/*c*	*P*-1
*a* (Å)	16.114(2)	4.8801(3)
*b* (Å)	6.7450(7)	5.6659(4)
*c* (Å)	7.5917(8)	15.1039(9)
*α* (°)	90	87.769(5)
*β* (°)	99.218(9)	83.162(5)
*γ* (°)	90	84.038(6)
*V* (Å^3^)	814.5(2)	412.26(5)
*Z*	2	1
*d*_calc_ (g cm^−3^)	1.612	1.736
*µ* (Mo K_α_) (mm^−1^)	1.096	1.235
*R*_int_	0.07	0.034
Refl. collected / unique	6838/1861	3137/1898
Refl. observed [*I* > 2*σ* (*I*)]/param./restr.	1265/123/0	1674/140/0
Completeness to *θ*_max_	0.999	0.998
*R*_1_, *wR*_2_ [*I* > 2*σ* (*I*)]	0.0516; 0.0849	0.0390; 0.0782
*R*_1_, *wR*_2_ (all data)	0.0850; 0.0979	0.0476; 0.0826
GOF on *F*^2^	1.02	1.052
Max. and min. residual density [e Å^−3^]	0.55/−0.47	0.42/−0.40

**Table 2:** Selected bond distances and angles in **1** and **2**.

1		2	
***Bond Distances*** **(Å)**			
C1–O1	1.246(3)	C1–O1	1.261(3)
C1–O2	1.264(3)	C1–O2	1.259(3)
Co1–O1	2.078(2)	Ni1–O1	2.051(2)
Co1–O1W	2.133(2)	Ni1–O1W	2.073(2)
Co1–O2 ^a^	2.068(2)	Ni1–O2W	2.032(2)
***Bond Angles*** **(°)**			
O1–C1–O2	124.6(3)	O1–C1–O2	126.5(2)
O1–Co1–O1W	85.94(9)	O1–Ni1–O1W	88.37(6)
O2 ^a^–Co1–O1	98.10(8)	O2W–Ni1–O1	87.35(7)
O2 ^b^–Co1–O1	81.90(8)	O2W–Ni1–O1W	90.91(7)
O1 ^c^–Co1–O1	180.0	O2W^d^–Ni1–O2W	180.0
O2 ^b^–Co1–O1W	89.09(9)	O2W–Ni1–O1 ^d^	92.65(7)
O2 ^a^–Co1–O1W	90.91(9)	O2W–Ni1–O1W ^d^	89.09(7)
O1 ^c^–Co1–O1W	94.06(9)	O1–Ni1–O1W ^d^	91.63(6)
O1W ^c^–Co1–O1W	180.0	O1^d^–Ni1–O1	180.0
O2 ^a^–Co1–O1 ^b^	180.0	O1W–Ni1–O1W ^d^	180.0

Symmetry codes: (a) *x*, −*y* + 1.5, *z* + 0.5; (b) −*x*, *y*−0.5, −*z* + 0.5; (c) −*x*, −*y* + 1, −*z* + 1; and (d) −*x* + 2, −*y* + 1, −*z*+1.

## Data Availability

The data underlying this article will be shared on reasonable request from the corresponding authors.

## Supplementary Materials

The following are available online at https://www.mdpi.com/article/10.3390/ma14133545/s1: Table S1. Geometries of hydrogen bonds and selected short contacts for studied structures; Figure S1. Powder X-ray diffraction patterns of 1: (a) experimental, (b) calculated; Figure S2. Powder X-ray diffraction patterns of 2: (a) experimental, (b) calculated; Figure S3. H-bond network (green lines) in compound 1. Symmetry codes: (a) x, −y+0.5, z+0.5; (b) –x, y−0.5, −z+0.5; (c) –x, −z+1, −z+1; (e) x, −y+0.5, z+0.5; Figure S4. H-bond network (green lines) in compound 2. Symmetry codes: (d) –x+2, −y+1, z+1; (f) –x+2, −y, −z+1; (g) –x+1, −y+1, −z+1; Figure S5. TG curves of 1 and 2 recorded in nitrogen atmosphere.
