# Supplementary material for: Structural and Thermal Investigations of Co(II) and Ni(II) Coordination Polymers Based on biphenyl-4,4′-dioxydiacetate Linker
Source: Materials (Basel). 2021 Jun 25;14(13):3545. doi: 10.3390/ma14133545 (PMC8269505; doi:10.3390/ma14133545)
Supplement: Supplementary file 1 [file materials-14-03545-s001.zip › materials-1246997-supplementary.pdf]

materials

Supplementary Materials

Structural and Thermal Investigations of Co(II) and Ni(II) Co-ordination Polymers Based on biphenyl-4,4′-dioxydiacetate Linker

Halina Głuchowska [1,*], Renata Łyszczek [1,*], Liliana Mazur [1] and Alexander M. Kirillov [2,3]

1 Department of General and Coordination Chemistry and Crystallography, Institute of Chemical Sciences, Faculty of Chemistry, Maria Curie-Skłodowska University in Lublin, M.C. Skłodowskiej Sq. 2, 20-031 Lublin, Poland; liliana.mazur@poczta.umcs.lublin.pl

2 Centro de Química Estrutural and Departamento de Engenharia Química, Instituto Superior Técnico, Universidade de Lisboa, Av. Rovisco Pais, 1049-001 Lisbon, Portugal; kirillov@tecnico.ulisboa.pt

3 Research Institute of Chemistry, Peoples' Friendship University of Russia (RUDN University), 6 Miklukho-Maklaya st., Moscow 117198, Russia

* Correspondence: halina.gluchowska@poczta.umcs.lublin.pl (H.G.); renata.lyszczek@poczta.umcs.lublin.pl (R.Ł.); Tel.: +48-81-537-57-40

Table S1. Geometries of hydrogen bonds and selected short contacts for studied structures.

Interaction	$d_{D\text{-}H}$ (Å)	$d_{H\cdots A}$ (Å)	$d_{D\cdots A}$ (Å)	<D-H···A (°)	Symmetry code
			Compound **1**		
O1W–H1W···O3^e	0.75(3)	2.40(3)	3.098(3)	157(3)	(e) x, -*y*+0.5, *z*+0.5
O1W–H2W···O1^e	0.94(5)	2.37(5)	3.005(4)	125(4)	
O1W–H2W···O2^c	0.94(5)	1.89(5)	2.737(4)	149(4)	(c) -*x*, -*y*+1, -*z*+1
C2–H2a···O1W^h	0.97	2.52	3.418(4)	153	(h) x, y, z-1
			Compound **2**		
O1W–H1W1···O2^f	0.77(3)	1.96(3)	2.700(3)	160(3)	(f) -*x*+2, -*y*, -*z*+1
O1W–H2W1···O1^g	0.79(3)	2.24(3)	2.851(3)	135(3)	(g) -*x*+1, -*y*+1, -*z*+1
O1W–H2W1···O3^g	0.79(3)	2.25(3)	2.979(3)	153(3)	
O2W–H2W2···O1W^g	0.87(3)	2.04(3)	2.823(3)	149(3)	
O2W–H1W2···O2^d	0.83(4)	1.88(4)	2.681(3)	163(3)	(d) -*x*+2, -*y*+1, -*z*+1
O2W–H2W2···O2W^g	0.87(4)	2.63(4)	3.052(3)	111(3)	(g) -*x*+1, -*y*+2, -*z*+1

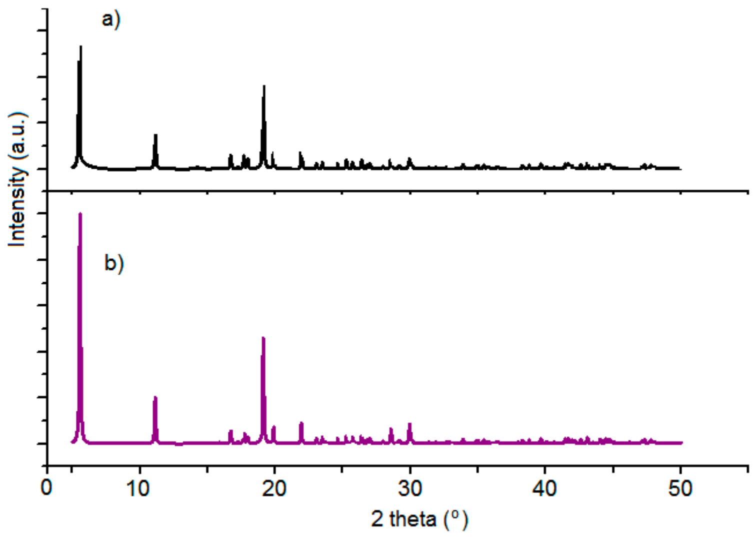

Figure S1. Powder X-ray diffraction patterns of **1**: (**a**) experimental, (**b**) calculated.

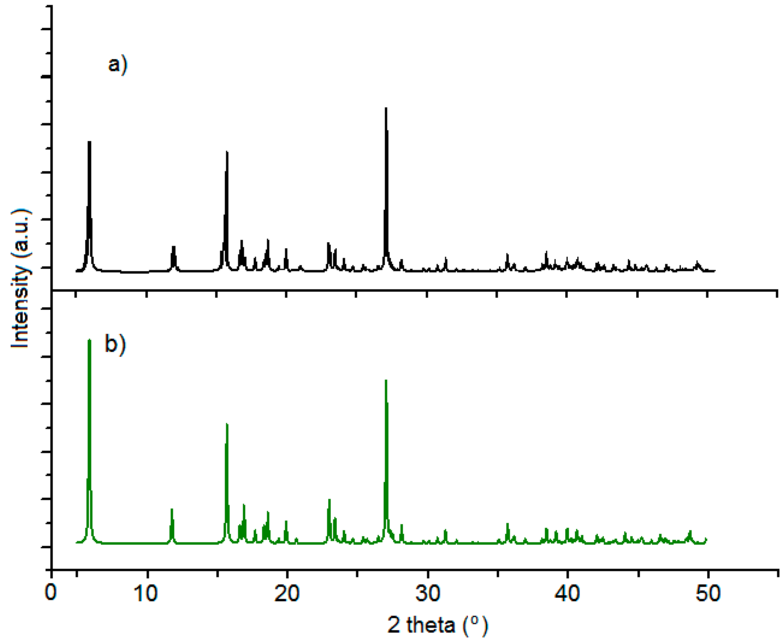

Figure S2. Powder X-ray diffraction patterns of **2**: (**a**) experimental, (**b**) calculated.

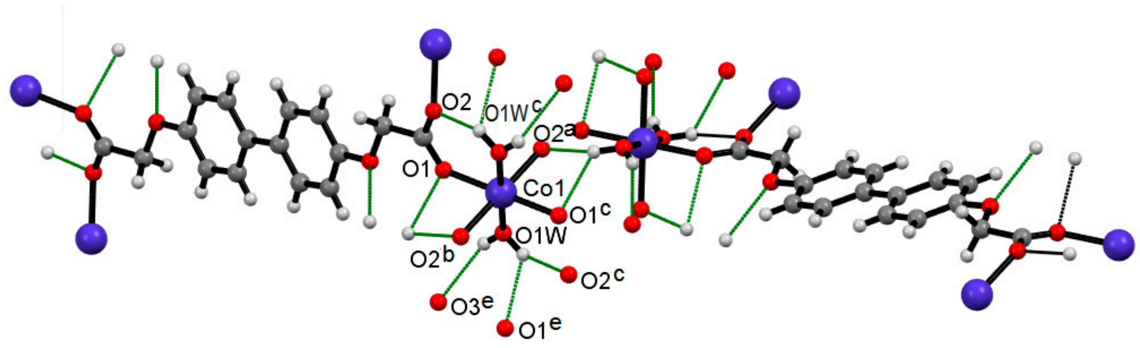

Figure S3. H-bond network (green lines) in compound **1**. Symmetry codes: (a) x, -y+0.5, z+0.5; (b) –x, y-0.5, -z+0.5; (c) –x, -z+1, -z+1; (e) x, -y+0.5, z+0.5.

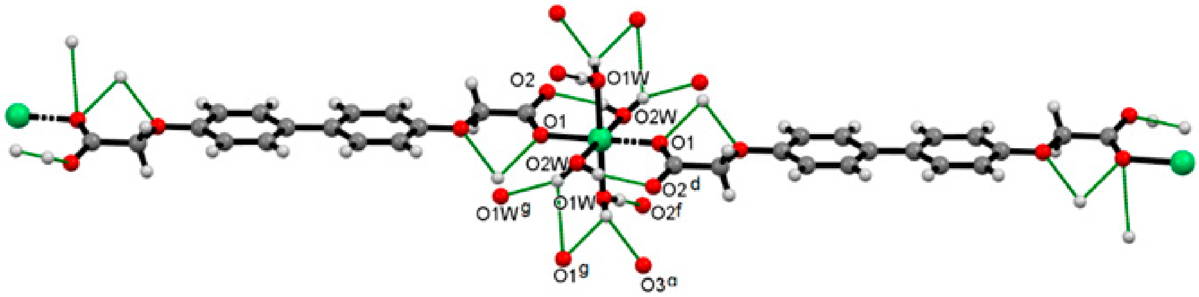

Figure S4. H-bond network (green lines) in compound 2. Symmetry codes: (d) –x+2, -y+1, z+1; (f) –x+2, -y, -z+1; (g) –x+1, -y+1, -z+1.

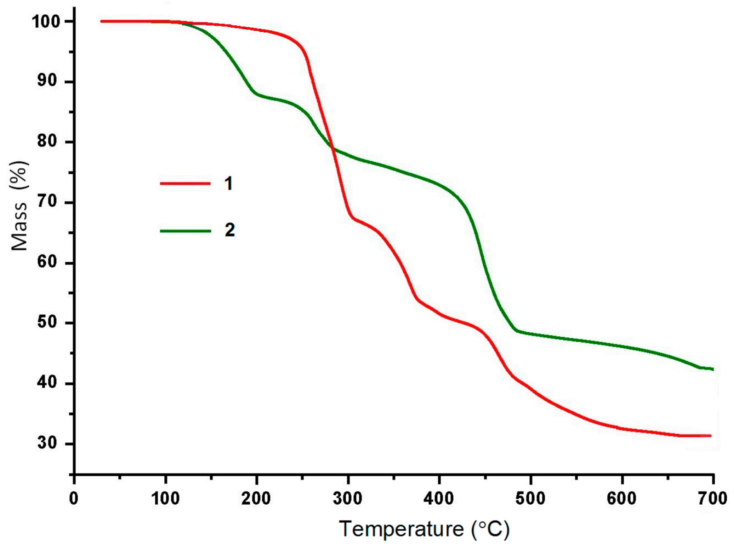

Figure S5. TG curves of **1** and **2** recorded in nitrogen atmosphere.
